# Best practices on blood and blood products for a prehospital hemorrhage protocol: consensus from the 2025 Canadian prehospital transfusion summit

**DOI:** 10.1186/s13049-026-01630-4

**Published:** 2026-06-03

**Authors:** Michael Peddle, Adam Greene, Jan Trojanowski, Andrew W. Shih, Eddie Chang, Susan Nahirniak, Dallas Pearson, Oksana Prokopchuk-Gauk, Doug Martin, John Froh, Charles Musuka, Cindy Seidl, Yulia Lin, Scott MacDonald, Lindsay Richards, Michael Farrell, Damien Miller, Johnathan Mack, Luis Da Luz, Ian R. Drennan, Kanwal Singh, Darshan Shingala, Brodie Nolan

**Affiliations:** 1 Ornge, Mississauga, ON Canada; 2 Division of Emergency Medicine, Schulich School of Medicine, Western University, London, ON Canada; 3 British Columbia Emergency Health Services, Vancouver, BC Canada; 4 School of Medicine, Cardiff University, Cardiff, Wales, UK; 5 Department of Pathology and Molecular Medicine, McMaster University, Hamilton General Hospital, Hamilton, ON Canada; 6 Shock Trauma Air Rescue Service, Calgary, AB Canada; 7 Faculty of Medicine, University of Alberta, Edmonton, AB Canada; 8 Alberta Precision Laboratories, Transfusion and Transplantation Medicine, Edmonton, AB Canada; 9 Shock Trauma Air Rescue Service, Saskatchewan, AB Canada; 10 Division of Transfusion Medicine, Department of Pathology and Laboratory Medicine, Saskatchewan Health Authority, Saskatoon, SK Canada; 11 College of Medicine, University of Saskatchewan, Saskatoon, SK Canada; 12 Shock Trauma Air Rescue Service, Manitoba, Canada; 13 Department of Transfusion Medicine, Shared Health Manitoba, Manitoba, Canada; 14 Precision Diagnostics and Therapeutics Program, Sunnybrook Health Sciences Centre, Toronto, ON Canada; 15 Department of Laboratory Medicine and Pathobiology, University of Toronto, Toronto, ON Canada; 16 Emergency Health Services LifeFlight, Halifax, Nova Scotia, ON Canada; 17 Nova Scotia Health Authority Provincial Blood Coordinating Team, Halifax, NS Canada; 18 Royal Canadian Medical Service, Canadian Armed Forces, Ottawa, ON Canada; 19 Transfusion Medicine and Hematology, Royal Victoria Hospital/McGill University Health Center, Montreal, Canada; 20 Canadian Blood Services, Ottawa, ON Canada; 21 Division of General Surgery, Sunnybrook Health Sciences Centre, Toronto, ON Canada; 22 FIRST60, Li Ka Shing Knowledge Institute, St. Michael’s Hospital, Toronto, ON Canada; 23 Department of Emergency Services and Sunnybrook Research Institute, Sunnybrook Health Sciences Centre, Toronto, ON Canada; 24 Defence Research and Development Canada, Toronto Research Centre, Toronto, ON Canada; 25 Department of Medicine, Yerevan State Medical University, Yerevan, Armenia; 26 Department of Emergency Medicine, St. Michael’s Hospital, Unity Health Toronto, 80 Bond Street, Toronto, ON M5B 1W8 Canada; 27 Division of Emergency Medicine, Department of Medicine, University of Toronto, Toronto, ON Canada; 28 Department of Emergency Medicine, University of British Columbia, Vancouver, BC Canada

**Keywords:** Prehospital, Transfusion, Trauma resuscitation, Massive hemorrhage protocol, Freeze-dried plasma, Whole blood, Fibrinogen concentrate, Critical care transport

## Abstract

**Background:**

Blood transfusion is increasingly recognized as a critical component of prehospital resuscitation. However, Canada’s vast geography, environmental extremes, and regional variation in health systems present unique challenges to standardized implementation of prehospital hemorrhage protocols (PHPs). The 2025 Canadian Prehospital Transfusion Summit (CPTS), convened by the Canadian Prehospital and Transport Transfusion (CAN-PATT) network, sought to develop consensus-driven best practices for prehospital transfusion strategies across diverse transport environments.

**Methods:**

A structured roundtable consensus process was held at CPTS 2025, bringing together over 50 subject matter experts from eight provinces, six different services/systems and a variety of operational roles to discuss the past, present and future direction of out-of-hospital blood transfusion in Canada. Key domains included: activation criteria, prioritization of blood components and adjuncts, product logistics, and rural/remote applicability. Recommendations were synthesized through iterative discussion, evidence review, and thematic analysis. Each recommendation was then voted upon until consensus was reached through all participants.

**Results:**

Consensus was reached on core components of a national PHP. Red blood cells and tranexamic acid were identified as foundational therapies. Freeze-dried plasma and whole blood offer logistical and clinical advantages, particularly in rural and remote areas, while fibrinogen concentrate and prothrombin complex concentrate are valuable adjuncts when plasma is unavailable. Calcium supplementation and active hypothermia prevention were emphasized as essential. Barriers to implementation included short product shelf-life, storage infrastructure, and regulatory constraints. The group recommended development of a standardized Canadian prehospital transfusion dataset and national data registry.

**Conclusions:**

The panel produced 12 consensus statements based on the available evidence and expert opinion including activation criteria, prioritization of blood products and adjuncts, product logistics, and rural/remote applicability. These consensus statements establish the basis for safe and effective PHP practices in Canada.

## Introduction

Timely access to blood products in the prehospital setting is a cornerstone of damage control and hemostatic resuscitation for patients experiencing traumatic hemorrhage [1–4]. The Canadian landscape presents unique challenges due to vast geographical distances, unpredictable weather, variable transport infrastructure, and resource disparities between urban and rural settings [5, 6]. Despite these barriers, significant advancements have been made by critical care transport organizations across the country, including the implementation of blood-on-board programs and use of hemostatic adjuncts [4, 7]. Although the term ‘out-of-hospital’ transfusion more accurately encompasses both direct-from-scene and interfacility transport settings, this paper uses the term ‘prehospital transfusion’ to include both contexts, with particular emphasis on blood-on-board programs rather than hospital-supplied blood.

Hemorrhagic shock remains a leading cause of preventable death in trauma [8, 9]. In-hospital massive hemorrhage protocols (MHPs) are structured systems designed to facilitate the rapid, coordinated delivery of blood products and adjunctive therapies [10]. MHPs operationalize balanced, ratio-based resuscitation while standardizing workflows, laboratory support, and product availability for patients with life-threatening bleeding [10–12]. The implementation of MHPs for trauma has been associated with improved patient outcomes, less overall blood utilization, and cost savings [13–16]. However, substantial logistical and operational challenges limit the direct translation of traditional in-hospital MHPs to prehospital and out-of-hospital environments. To better reflect the unique clinical and operational considerations in this setting, the term prehospital hemorrhage protocol (PHP) has been proposed to describe a structured bundle of interventions designed for the early recognition and treatment of hemorrhagic shock prior to hospital arrival. PHPs adapt core principles of hospital-based massive hemorrhage protocols to the constraints of prehospital care and include the early administration of blood products and hemostatic adjuncts, along with measures aimed at mitigating the “lethal diamond” of trauma—coagulopathy, hypothermia, acidosis, and hypocalcemia. Despite ongoing advancements in trauma resuscitation, significant gaps persist in the standardization and availability of blood components and products across Canada’s prehospital systems [4, 5, 7]. Currently, there is no national framework guiding prehospital transfusion practices, resulting in varied implementation across provinces and jurisdictions. Additionally, operational challenges such as limited cold storage capabilities, short product shelf life (e.g., thawed plasma), and inconsistent use of clinical decision tools all contribute to the lack of standardization of prehospital transfusion ‘best practices’. This fragmented landscape underscores the urgent need for standardized guidance, optimized logistics, and equitable access across urban and rural transport environments.

## Methods

The Canadian Prehospital and Transport Transfusion (CAN-PATT) network was established in 2021 to collaborate, standardize, and evaluate the effectiveness of out-of-hospital blood transfusion (OHBT) across Canada [5]. A summary of the critical care transport organizations involved in CAN-PATT are presented in Table 1.

**Table 1:** Overview of critical care transport organizations in CAN-PATT

Organization	Service Area Population	Geography covered (km sq)	Annual number of calls	Total bases (*n*)	Median Transport Time	Annual blood-on-board transfusions	Bases with blood-on-board program	Number of coolers per base	Blood products per cooler
Ornge	14,500,000	1,076,395	20,000	14	**RW**: 46 min **FW**: 64 min **Land**: 45 min	150 patients	4	2 to 4	2 RBCs
BCEHS	5,000,000	944,000	8,300	7	**RW**: 29 min FW: *	50 patients	3	2 (Vancouver) 1 (Nanaimo, Kamloops)	2 RBC + 2 Plasma (Vancouver) 2 RBC (Nanaimo, Kamloops)
STARS Alberta	6,000,000	595,000	1,450	3	**RW**: 36 min	100 patients	3	1	2 RBCs
STARS Saskatchewan	1,180,000	586,710	1,500	3	**RW**: 43 min **FW**: 90 min	50 patients	3	2	2 RBCs per cooler + 3rd cooler with Fibrinogen Concentrate and PCC
STARS Manitoba	1,300,000	649,950	1,300	2	**RW**: 30 min **FW**: 85 min	60 patients	2	3	2 RBCs per cooler + 3rd cooler with PCC
EHS LifeFlight	1,923,000	133, 850	1,500	1	**RW**: 35 min **FW**: 40 min **Land**: 90 min	20 patients	1	2	2 RBCs

BCEHS: British Columbia Emergency Health Services, STARS: Shock Trauma Air Rescue Service, EHS: Emergency Health Services, RW: rotor-wing, FW: fixed-wing, RBC: red blood cells, PCC: prothrombin complex concentrate, *: data unavailable

In June 2025, CAN-PATT held the inaugural Canadian Prehospital Transfusion Summit (CPTS), bringing together over 50 subject matter experts from eight provinces, six different services/systems and a variety of operational roles including emergency, trauma and transfusion medicine physicians, critical care paramedics, registered nurses and transfusion medicine specialists to discuss the past, present and future direction of prehospital blood transfusion in Canada. The meeting was chaired by BN and MP, and participants were invited based on geographic and subject matter diversity. The meeting was held in-person with virtual attendance optional for those unable to attend in person. Due to the closed nature of the larger CPTS 2025 summit, there was no patient or public participants involved in this study. A literature review on massive hemorrhage protocols, current hemorrhagic shock treatments, and ongoing clinical trials was done by MP and BN and discussed throughout the larger CPTS 2025 meeting. Using a structured roundtable format, participants identified shared priorities and offered pragmatic recommendations to build consensus on the essential components of an optimal prehospital hemorrhage protocol. The discussion examined key domains including activation criteria, prioritization of blood products and components, and the role of hemostatic adjuncts. Using a structured roundtable format, participants identified shared priorities and practical recommendations. Recommendations were synthesized through in-person iterative discussion, evidence review, and thematic analysis. Final recommendations were then continuous refined and voted upon (agree/disagree) in real time until 100% consensus was obtained by the participants. As this was a roundtable exercise, voting was not anonymous.

## Results

### Prediction scores and tools for PHP activation

The use of predictive scores to initiate a massive hemorrhage protocol has been recommended for use in Ontario’s provincial in-hospital massive hemorrhage protocol guidelines, however the utility and use of these scores in the prehospital environment is variable [10, 17]. Various clinical scoring tools have been developed, such as the Assessment of Blood Consumption (ABC) score and shock index (SI) [17, 18]. The ABC score incorporates systolic blood pressure, heart rate, focused assessment with sonography in trauma (FAST), and mechanism of injury [11, 19]. Shock index, calculated as heart rate divided by systolic blood pressure, offers a rapid bedside marker of hypoperfusion [20]. At CPTS 2025, it was highlighted that point of care ultrasound and the ability to perform a FAST exam in the prehospital environment was not standard for most Canadian critical care transport organizations, limiting the application of many derived scores that predict massive blood loss. Nova Scotia LifeFlight highlighted their routine monitoring of the shock index before and after transfusion to evaluate response and guide ongoing therapy. Several programs, including STARS and Ornge, base transfusion activation on integrated assessments by experienced flight crews and transport physicians, supplemented by physiological markers and trauma triage criteria. Overall, clinical judgement remains paramount.

#### Panel recommendation

The panel strongly recommends the development of an out-of-hospital specific prediction tool or score that could more easily identify patients who may require some blood products, as opposed to many of the current tools or scores that were derived to identify patients who require a massive transfusion (10 or more units of blood products).

### Red blood cells

Red blood cells (RBCs) are foundational in hemorrhagic shock resuscitation, restoring oxygen-carrying capacity and intravascular volume [21–23]. Most Canadian critical care transport programs carry type O, RhD-negative RBCs on board, with most organizations stocking two units per mission in temperature-controlled coolers. There was consensus at CPTS 2025 that clinical evidence and experience suggest early prehospital transfusion improves outcomes in trauma patients with ongoing hemorrhage, particularly when transport times are prolonged [22, 24]. There is also limited or no availability of RBCs at many small hospitals and nursing stations in rural Canada, creating additional challenges in early blood product administration for many bleeding patients [25]. Furthermore, the panel recognizes that blood-on-board programs improve access to care for rural, remote, and indigenous communities across Canada.

#### Panel recommendation

The panel recommends at least two units of group O RhD-negative RBC be included in any PHP.

### Thawed plasma

Thawed plasma is critical in correcting coagulopathy and providing volume expansion. However, its use in the prehospital environment is hampered by significant logistical constraints [26]. Once thawed, plasma has a shelf life of only five days, resulting in high wastage rates when unused. Most blood-on-board programs in Canada are not hospital-based, requiring the plasma to be pre-packaged and forward deployed to the critical care transport base, where they are rotated in-and-out of the hospital system every 2–3 days. This leaves little time for hospitals to use thawed plasma returned from blood-on-board programs. The British Columbia Emergency Health Services (BCEHS) Air Ambulance and Critical Care Operations is one of a few programs to routinely bring thawed plasma to the point of injury and has reported a 13.6% wastage rate. Likewise, Ornge in Ontario found that there was a doubling of wastage of thawed plasma at the supporting blood bank just by adding thawed plasma to the blood-on-board program during participation in the Canadian Study of Whole blood in Frontline Trauma (SWiFT Canada). This issue is compounded by limited in-hospital demand, preventing reintegration of unused product into the in-hospital supply chain. The experience of Sunnybrook, shared at CPTS 2025, illustrated the challenge: when prehospital coolers containing thawed plasma return with only 24–48 h of shelf life remaining, they are often wasted due to limited in-hospital use.

#### Panel recommendation

The panel agrees that while the early administration of TP has demonstrated select mortality benefit in patients with blunt trauma, moderate transfusion requirements, traumatic brain injury, certain cytokine expression profiles and/or when transport times were greater than 20 min, ongoing logistical challenges have limited its widespread adoption in Canada.

### Freeze-dried plasma

Freeze-dried plasma (FDP) is an emerging alternative to thawed plasma, particularly in settings where cold chain maintenance and wastage are significant barriers [27, 28]. FDP is shelf-stable for two years at room temperature and can be reconstituted rapidly with sterile water at the point of care [28]. These features make it highly suitable for prehospital, rural, and military use.

Defence Research and Development Canada, in collaboration with the Canadian Armed Forces, has extensively evaluated the biophysical and haemostatic properties of FDP. Laboratory data confirm that reconstituted FDP preserves clotting factor function, is comparable to fresh frozen plasma in PT/aPTT values and remains effective even after multiple freeze-thaw cycles [28]. At CTPS 2025, researchers from Defence Research and Development Canada presented data on arctic field testing, demonstrating FDP’s resilience to extreme environmental conditions, although challenges persist in preventing the freezing of water used in reconstitution (unpublished data). Clinical adoption in Canada remains limited due to regulatory barriers, with FDP not yet approved by Health Canada. However, international experience (particularly from France, Germany, and Israel) demonstrates its safety and efficacy in both civilian and combat trauma.

#### Panel Recommendation

The panel strongly recommends the integration of FDP into any PHP program, especially in “blood deserts” where traditional products are inaccessible.

### Platelets

Platelets are integral to hemostasis and are a component of balanced transfusion strategies and many in-hospital massive hemorrhage protocols [10]. However, their short shelf life (5–7 days) and unique storage requirements (constant agitation at room temperature) make them unsuitable for the out-of-hospital environment. The lack of deployable platelet products presents a gap in comprehensive prehospital transfusion resuscitation. It was noted that there is ongoing work in cold-stored and lyophilized platelet substitutes, but these are not yet in operational use.

#### Panel recommendation

The panel agrees the routine use of platelets should not be part of any PHP.

### Whole blood

Whole blood simultaneously provides RBCs, plasma, and platelets in a single unit, simplifying logistics while replicating physiological ratios, and observational and ex-vivo studies have shown potential hemostatic benefit compared to component therapy [26, 29–31]. The SWIFT Canada trial, Canada’s first randomized controlled study of prehospital whole blood, compared whole blood to RBC + thawed plasma administration in trauma patients. Additionally, STARS Manitoba was a participating site in the TOWAR (The Type O whole blood and assessment of AGE during prehospital resuscitation trial), which compared prehospital whole blood to standard care (which could be component therapy or crystalloid fluids). Early findings presented at CPTS 2025 suggest there is feasibility and safety of whole blood in the Canadian prehospital environment (unpublished data). It was discussed however that widespread implementation may be constrained by limited group O low-titre donors, logistical complexities for pan-Canadian deployment, and shorter shelf-life of whole blood compared to RBCs (currently whole blood in Canada has a shelf-life of 21 days, compared to 42 days for RBC). It was also discussed that the evidence for whole blood outside of trauma (i.e., for obstetrical or gastrointestinal bleeding) is very limited. Thus, whole blood may not be the right product to carry for all critical care transport organizations or bases, depending on the nature of bleeding.

#### Panel recommendation

While whole blood appears to be the emerging primary blood product used in prehospital trauma resuscitation, randomized clinical trial evidence is still lacking, and both practical limitations and bleeding etiology case mix may limit its pan-Canadian adoption.

### Prothrombin complex concentrate

Prothrombin Complex Concentrate (PCC) contains vitamin K-dependent clotting factors (II, VII, IX, X, Protein C and S) and is effective in reversing warfarin anticoagulation and treating coagulopathy. PCC showed superiority in comparison to plasma, with better hemostatic responses and reduced transfusion needs in the context of adult cardiac surgery patients with coagulopathic bleeding [32]. It is increasingly being considered in trauma, especially in settings where blood products are unavailable or delayed [10, 33]. PCC’s room temperature stability and rapid reconstitution make it especially well-suited for rural and prehospital environments where cold chain infrastructure is limited. Some programs, including STARS in Saskatchewan and Manitoba, include PCC in their PHP. Alberta, however, has expressed caution due to concerns over thromboembolic risk [34]. Evidence from European trauma systems suggests potential benefit when PCC is used selectively and guided by diagnostic tools [35]. Results of the FiiRST-2 trial, a multicentre randomized clinical trial conducted in Canada were presented at CPTS 2025^33^. FiiRST-2 evaluated the pre-emptive use of Fibrinogen Concentrate combined with PCC as a first-line in-hospital massive hemorrhage protocol intervention, compared to standard plasma therapy in bleeding trauma patients. Although halted early due to futility, the study confirmed safety (no difference in thromboembolic complications) and showed comparable efficacy outcomes with plasma (similar transfusion rates within the first 24 h). However, participants acknowledged further investigation in a larger trial is warranted. Experts discussed some of the practical challenges in reconstitution of PCC with multiple vials, especially in an austere environment with limited personnel available.

#### Panel recommendation

The panel supports the use of PCC as a substitute for plasma if it is not available as part of a PHP, however this is based on limited evidence.

### Fibrinogen concentrate

Fibrinogen is the first coagulation factor depleted in trauma-induced coagulopathy [36, 37]. Fibrinogen Concentrate (FC) offers a rapid, pathogen-reduced alternative to cryoprecipitate, and is increasingly favoured due to its logistical and clinical advantages. Unlike cryoprecipitate, FC does not require thawing, group-matching, or pooling from donors. FC allows for faster administration and reduced risk of transfusion-related complications. It is also easier to store, reconstitute, and to provide precise dosing, making it suitable for prehospital and rural environments. Additionally, cryoprecipitate has been phased out from the Canadian blood supply since the results of the FIBRES trial showing non-inferiority of FC compared to cryoprecipitate [38]. The FiiRST-2 data supported the continued use of FC and PCC where plasma is not available [33]. CPTS experts also addressed the results of CRYOSTAT-2 trial, which found that among trauma patients who required in-hospital massive hemorrhage protocols, the addition of early and empirical high-dose cryoprecipitate to standard care did not improve all cause 28-day mortality [39]. It was noted that although guidelines now recommend routine testing prior to FC administration, prehospital measurement of FC is not available nor feasible in most of the Canadian prehospital or rural environments.

#### Panel recommendation

The panel suggests that FC may be a reasonable addition to a PHP after the initial blood components have been administered if bleeding is ongoing, or in combination with PCC if plasma is not available.

### Tranexamic acid

Tranexamic Acid is an antifibrinolytic agent used across multiple clinical settings for its ability to provide hemostatic control and improve clinical outcomes in patients that are bleeding. Evidence suggests a modest mortality benefit when used for trauma resuscitation, but it’s utility and efficacy in other clinical scenarios is less clear and requires clinical judgement. While the optimal dosing in emergency settings is unclear, emerging evidence suggest that a 2g IV bolus (instead of the traditional 1g IV bolus + 1g IV infusion) might be more convenient for providers without significant differences in mortality, functional outcomes, or adverse events [40]. TXA is widely used in Canadian prehospital trauma care, with administration occurring early during transport. Most services report high compliance, and many critical care transport organizations have incorporated TXA into standard protocols with an upfront dose of 2g IV over 10–20 min.

#### Panel recommendation

The panel recommends the adoption of a 2g IV bolus dosing strategy for the out-of-hospital administration of TXA.

### Calcium

Calcium plays a critical role in clotting cascade activation and myocardial function [2, 41]. Hypocalcemia is common during massive transfusion due to citrate binding from blood products. In hospital massive hemorrhage protocol guidelines recommend monitoring ionized calcium and supplementing as needed during massive hemorrhage protocol activation [10]. Despite this, to date, there is no high-quality evidence investigating the role of calcium supplementation in trauma patients receiving blood transfusions. All studies are retrospective analyses and are therefore subject to confounding and information bias [41]. Many critical care transport organizations also carry point-of-care blood analysers which can provide a point-of-care ionized calcium. Several critical care transport organizations include calcium gluconate or calcium chloride in their PHP, with protocols guiding administration based on transfusion volume or a measure ionized calcium level.

#### Panel recommendation

The panel recommends routine calcium supplementation, with either calcium chloride 1g IV or calcium gluconate 3g IV, to be given at regular intervals during PHP and considered at minimum after a second unit of blood is transfused. When possible, targeted calcium replacement should be performed when ionized calcium levels are low.

### Hypothermia adjuncts

Hypothermia is a major cause of morbidity and mortality in trauma and massive hemorrhage [42, 43]. Prevention and treatment of hypothermia early in bleeding patients is essential to avoid worsening of acidosis and coagulopathy. Blood product warming, active rewarming devices, and environmental protection measures (e.g., thermal blankets, warmed fluids) are employed by most critical care transport teams. The STAY WARM trial, was a pilot study in Canada that demonstrated the feasibility of using self-warming chemical blankets in bleeding trauma patients on admission to the trauma bay [42]. The STAYWARM-2 trial (ongoing) is investigating differences in temperature across the first 8 h post admission in bleeding trauma patients using chemical blankets compared with conventional warmed flannel blankets. The use of chemical blankets in the prehospital setting may provide better temperature control. CPTS 2025 participants discussed interest in integration of this low-cost, scalable solution and how they could form a critical element of bundled care for hemorrhagic shock in this setting.

#### Panel recommendation

The panel strongly recommends the prevention and mitigation of hypothermia to be a cornerstone of any PHP.

### Quality assurance and recommendations for best practice

The roundtable revealed strong consensus on several key themes for improving prehospital trauma transfusion in Canada (Table 2). While implementation strategies vary regionally, shared priorities emerged around early blood administration, streamlined adjunct use, and feasible warming strategies.

**Table 2:** National framework vision for prehospital transfusion best practices

1) Develop a national prehospital hemorrhage protocol guideline on when and how to administer RBCs, FDP, FC, PCC, calcium, TXA, and hypothermia adjuncts.
2) Support the standardization of prehospital hemorrhage protocol activation based on clinical signs and validated decision tools, rather than patch-dependent physician authorization alone.
3) Create a national registry for transfusion practices to monitor utilization, safety, and outcomes, which will guide continuous quality improvement and policy decisions.
4) Leverage ongoing trials (i.e. STAY WARM-2) to integrate practical, scalable hypothermia management solutions into standard trauma bundles.
5) Continue collaboration through national networks (i.e. CAN-PATT) with regular meetings to review emerging practices and new evidence.
6) Generate Canadian specific data on prehospital transfusion practices across the country, including pan-Canadian prehospital transfusion clinical trials (i.e. SWiFT Canada 2.0)

A ‘critical threshold’ strategy was widely supported: FC/PCC are administered after 2–4 units of RBC if hypotension persists and no plasma or coagulopathy testing is available. Simulations and upfront training are considered crucial to successful implementation, particularly in remote and resource-limited settings. Likewise, calcium administration was broadly practiced either with the first or second unit and then repeated at regular intervals, guided by ionized calcium measurements where feasible. For hypothermia, chemical warming blankets and in-line fluid warmers were considered essential.

Across all domains, the lack of national standards was repeatedly cited as a barrier to quality and equity. Participants unanimously endorsed creation of a national registry to track transfusion utilization, adverse events, and clinical outcomes linked to in-hospital data. This would enable benchmarking, guide iterative improvement, and inform regulatory evolution for critical care transport organizations.

#### Panel recommendation

The panel strongly recommends the creation of a comprehensive national registry to guide quality assurance and quality improvement in Canada.

### Strengths and limitations

Although these recommendations were developed within the context of the Canadian healthcare system, many of the challenges described—such as long transport times, limited blood product availability, and logistical constraints of prehospital transfusion—are shared by trauma systems internationally. As such, this consensus process may serve as a practical example of how multidisciplinary stakeholders can collaboratively develop standardized protocols for prehospital hemorrhage management. The framework outlined here may therefore inform similar policy development and protocol implementation efforts in other jurisdictions seeking to expand or standardize prehospital transfusion practices.

## Conclusion

The panel produced 12 consensus statements based on the available evidence, shared experiences and expert opinion regarding activation criteria, prioritization of blood products and adjuncts, product logistics, and rural/remote applicability. These consensus statements establish the basis for safe, effective and standardized OHBT practices in Canada.

## Data Availability

Data sharing is not applicable to this article as no datasets were generated or analysed during the current study.

## Declarations

### About this supplement

This article has been published as part of Scandinavian Journal of Trauma, Resuscitation and Emergency Medicine Volume 34 Supplement 1, 2026: Proceedings of the Canadian Prehospital Transfusion Summit. The full contents of the supplement are available online at https://link.springer.com/journal/13049/volumes-and-issues/34-1/supplement.
